# *Cupriavidus* sp. HN-2, a Novel Quorum Quenching Bacterial Isolate, is a Potent Biocontrol Agent Against *Xanthomonas campestris* pv. *campestris*

**DOI:** 10.3390/microorganisms8010045

**Published:** 2019-12-25

**Authors:** Tian Ye, Tian Zhou, Qiting Li, Xudan Xu, Xinghui Fan, Lianhui Zhang, Shaohua Chen

**Affiliations:** 1 State Key Laboratory for Conservation and Utilization of Subtropical Agro-bioresources, Guangdong Laboratory of Lingnan Modern Agriculture, Integrative Microbiology Research Centre, South China Agricultural University, Guangzhou 510642, China; 2 Guangdong Province Key Laboratory of Microbial Signals and Disease Control, South China Agricultural University, Guangzhou 510642, China

**Keywords:** diffusible signal factor, *Cupriavidus* sp., degradation activity, biocontrol, quorum sensing, *Xanthomonas campestris* pv. *campestris*

## Abstract

Diffusible signal factor (DSF) represents a family of widely conserved quorum sensing (QS) signals involved in the regulation of virulence factor production in many Gram-negative bacterial pathogens. Quorum quenching, which disrupts QS either by degradation of QS signals or interference of signal generation or perception, is a promising strategy for prevention and control of QS-mediated bacterial infections. In this study, a novel DSF-degrading strain, HN-2, was isolated from contaminated soil and identified as *Cupriavidus* sp. The isolate exhibited superior DSF degradation activity and completely degraded 2 mmol·L^–1^ of DSF within 24 h. Analysis of the degradation products of DSF by gas chromatography–mass spectrometry led to the identification of trans-2-decenoic acid methyl ester as the main intermediate product, suggesting that DSF could be degraded by oxidation and hydroxylation. Moreover, this study presents for the first time, evidence that *Cupriavidus* sp. can reduce the black rot disease caused by *Xanthomonas campestris* pv. *campestris* (*Xcc*). Application of the HN-2 strain as a biocontrol agent could substantially reduce the disease severity. These findings reveal the biochemical basis of a highly efficient DSF-degrading bacterial isolate and present a useful agent for controlling infectious diseases caused by DSF-dependent bacterial pathogens.

## 1. Introduction

**Figure 1:**
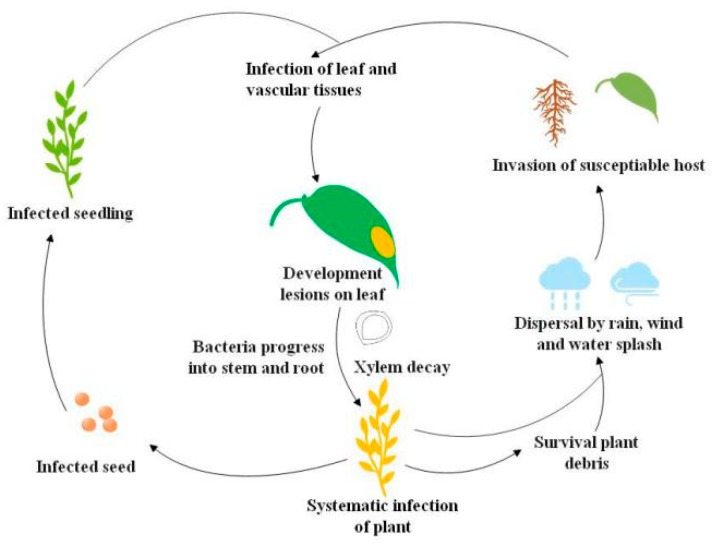
Life cycle of the black rot pathogen *Xanthomonas campestris* pv. *campestris* (*Xcc*) [5].

Black rot pathogen *Xanthomonas campestris* pv. *campestris* (*Xcc*) has a wide host range but mainly infects cruciferous crops. Economically important cruciferous crops are consistently damaged by the black rot pathogen, and the death of plant tissues by *Xcc* infection leads to severe crop losses [1,2]. Black rot epidemiological studies revealed that black rot pathogen thrives in warm, humid climates and rapidly disseminates in the field to infect healthy crops [3]. The *Xcc* inoculum spreads from the infected seed to the epicotyl of healthy seeds, and as a result, cotyledons develop blackened margins and then shrivel and drop [1,4]. Bacteria propagate in young stems and leaves through the vascular system, and the disease appears as V-shaped chlorotic to necrotic lesions on leaf margins. During humid conditions, bacteria in guttation droplets are spread to neighboring plants by wind, rain, water splashes, and mechanical equipment [1,4]. Hydathodes, leaf wounds by insects, and roots are the main *Xcc* entry points in plants, but occasionally, infection also occurs through stomata [1]. Systemic *Xcc* infection in the host occurs from hydathodes of leaf margins into the plant vascular system and ultimately invasion of suture veins, which leads to the production of *Xcc*-infected seeds. *Xcc* can survive in soil plant debris for up to two years, although for not more than six weeks in free soil [1]. Long-distance *Xcc* transmission can also happen through flying insect vectors [5] (Figure 1).

**Figure 2:**
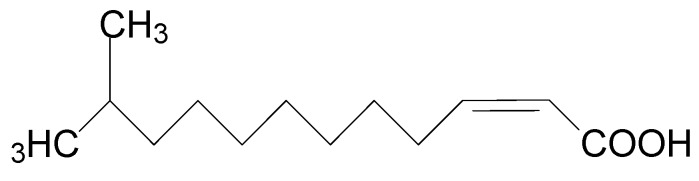
Chemical structure of diffusible signal factor (DSF).

Diffusible signal factor (DSF) is structurally characterized as cis-11-methyl-2-dodecenoic acid (Figure 2) and is widely conserved in many Gram-negative bacterial pathogens such as *Xcc*, *Xanthomonas oryzae* pv. *oryzae* (*Xoo*), *Stenotrophomonas maltophilia*, *Burkholderia cepacia*, and *Pseudomonas aeruginosa* [6]. Accumulation of DSF activates the genes related to *Xcc* tissue-macerating pathogenicity [7]. Quorum sensing (QS) is the regulation of virulence factors at high population densities through cell–cell communication, and it inhibits premature activation of plant defense for successful infection [8,9]. QS can be simply understood as bacterial communication via the DSF [6,7]. QS regulates virulence factors including extracellular pectinolytic and cellulolytic enzymes, pectinases, lyases, xylanases, and cellulases, which contribute to the degradation of plant cell walls [10,11,12,13]. Disruption of QS may prevent the production of *Xcc* virulence factors, which makes it an appropriate target for the development of new phyto-protective agents [14].

Recently, exploration of bacteria for the biological control of plant pathogens has gained the keen interest of researchers. Inactivation of cell-to-cell signaling compounds, such as *N*-acyl homoserine lactone (AHL) and DSF, by bacterial degradation disrupts QS, a phenomenon known as quorum quenching (QQ) [15]. The first QQ bacteria were isolated from soil and identified as Gram-positive *Bacillus* sp. [16]. AHL-degrading bacteria have been reported to significantly decrease the virulence of the soft rot potato pathogens *Pectobacteria atrosepticum* and *Pectobacteria carotovorum* [15,17,18,19]. In contrast to other antimicrobial methods, this strategy efficiently attenuates the virulence without eradicating the pathogen [20,21]. However, studies on the QQ mechanism of DSF-degrading bacteria as an effective biocontrol approach are sparse in the literature. DSF degraders and their molecular mechanism and parameters for attenuating *Xcc* virulence have received little attention.

Herein, a novel DSF-degrading bacterial strain, *Cupriavidus* sp. HN-2, was identified as exhibiting superior DSF-degrading activity. Moreover, application of the strain HN-2 as a biocontrol agent could significantly reduce the black rot disease severity caused by *Xcc*. These results suggest that the HN-2 strain possesses promising potential that can be further expanded for better control of DSF-dependent pathogens and protection of plants from the respective infectious diseases.

## 2. Materials and Methods

### 2.1. Chemicals and Plants

DSF (≥99%) was obtained from Shanghai UDChem Technology Co., Ltd (Shanghai, China). The chemical was dissolved in methanol at a stock concentration of 100 mmol·L^–1^ and stored in dark bottles at 4 °C prior to use. Working solutions were made by diluting stock with culture medium and filtering it through a 0.2 μm membrane. The pure active ingredient of the pesticide, streptomycin (98%), was used as a positive control at 3.3 g·mL^–1^. Radishes (*Raphanus sativus* L.) were purchased from a local market (Guangzhou, China), and healthy plants were selected for the experiments.

### 2.2. Bacterial Strains and Culture Conditions

Black rot pathogen *Xanthomonas campestris* pv. *campestris* (*Xcc*), strain XC1, and *Escherichia coli* DH5α were stocked in the laboratory. *Xcc* strains were maintained in Luria Bertani (LB) (composition g·L^–1^): yeast extract 5.0, tryptone 10.0, NaCl 10.0) with Rifampicin (30 μg·mL^–1^). Additionally, *E. coli* strains were grown in LB at 37 °C.

### 2.3. Isolation of DSF-Degrading Bacteria from Soil Samples

Soil samples were collected from the agricultural contaminated fields of the South China Agricultural University in July 2016. Approximately 300 g of soil were collected from the upper layer (5–10 cm), and samples were stored at 4 °C after collection. DSF-degrading bacteria were isolated using an enrichment procedure. To isolate DSF-degrading bacteria, 5 g soil samples were added to a minimal salt medium (MSM) (composition (g·L^–1^): (NH_4_)_2_SO_4_ 2.0, Na_2_HPO_4_·12H_2_O 1.5, KH_2_PO_4_ 1.5, MgSO_4_·7H_2_O 0.2, CaCl_2_·2H_2_O 0.01, FeSO_4_·7H_2_O 0.001, pH 7.2) supplemented with DSF at a concentration of 1 mmol·L^–1^ and cultivated for 7 d at 30 °C and 200 rpm. After 7 d, the suspension was transferred to another MSM containing DSF (2 mmol·L^–1^) at 10% inoculum and cultivated under the same conditions for 7 d. The procedure was repeated until the DSF concentration increased to 4 mmol·L^–1^. The final suspension was serially diluted (10^−1^ to 10^−8^) and spread on LB agar plates. After incubation at 30 °C for 24–72h, colonies with different characteristics were selected and transferred to fresh medium. The procedure was repeated until a pure culture was obtained.

For the screening of the bacterial isolates, isolates were grown in MSM containing 2 mmol·L^–1^ DSF as the sole source of carbon for 24 h at 30 °C and 200 rpm. After 24 h, cultures were centrifuged, and the remaining DSF was extracted from the supernatant. The remaining amount of DSF was determined by high performance liquid chromatography (HPLC). The bacterial strain HN-2 was isolated from the soil samples and selected for further studies based on its effective DSF degradation.

### 2.4. Identification of Strain HN-2

The morphology of strain HN-2 was studied by inoculating it on an LB medium for 24 h at 30 °C. The colony and cellular morphologies of strain HN-2 were investigated under an electron microscope (BH-2 Olympus, Tokyo, Japan) and a scanning electron microscope (XL-30 ESEM, Philips Optoelectronics Co., Ltd., Amsterdam, Holland) on LB plates and slides, respectively.

Bacterial strain HN-2 was also identified by partial sequence analysis of cloned 16S rDNA genes. The strain was grown on LB, and PCR amplification of DNA was performed by using a 16S rDNA universal bacterial primer set 27 F (5′-AGAGTTTGATCCTGGCTCAG-3′) and 1492 R (5′-GGTTACCTTGTTACGACTT-3′). PCR conditions were as follows: initial denaturation at 94 °C for 5 min, 32 cycles of denaturation at 94 °C for 30 s, annealing at 55 °C for 30 s, and an extension at 72 °C for 1 min. The reaction followed a final extension at 72 °C for 5 min. The PCR product was sequenced, and a homology search was carried out using BLAST (Basic Local Alignment Search Tool) (https://blast.ncbi.nlm.nih.gov/Blast.cgi) at the National Center for Biotechnology Information (NCBI) (https://www.ncbi.nlm.nih.gov) [22]. Sequences with close similarity were used for the identification and phylogenetic analysis of strain HN-2.

### 2.5. DSF Degradation Assays

To study the relationship between DSF degradation and growth of isolate HN-2, DSF was added to cell suspensions of test strains, and the remaining DSF was detected with HPLC at different time intervals. Growth of HN-2 was calculated by using an ultraviolet spectrophotometer (GeneTech (Shanghai) Co., Ltd., Shanghai, China) at an optical density of 600 nm (OD_600_). The methodology was comprised of different steps as follows: HN-2 isolate was inoculated into 5 mL LB and incubated overnight at 30 °C and 200 rpm. Overnight cultures were added to 50 mL MSM medium containing 2 mmol·L^–1^ DSF and incubated at 30 °C for 48 h. Controls consisted of 50 mL MSM medium containing 2 mmol·L^–1^ DSF. DSF was extracted three times from the supernatant with an equal volume of ethyl acetate. The mixture was dried in a rotary evaporator (Synthware, Beijing, China) and re-dissolved in 2 mL methanol. DSF was subsequently extracted and quantified at various incubation time intervals. Growth of HN-2 was also measured synchronously.

The extracted samples were subjected to HPLC with a C_18_ reverse-phase column and eluted with methanol and water (80:20, *v*/*v*, water containing 0.1% formic acid) at a flow rate of 1 mL·min^–1^. A diode array detector (Alliance e2695, Waters Corporation, Milford, MA, USA) was used for monitoring at the UV wavelength of 210 nm.

### 2.6. Identification of DSF Degradation Products

To identify DSF and its degradation products, the HN-2 strain was grown in 100 mL MSM medium containing 2 mmol·L^–1^ of DSF. Non-inoculated samples containing the same amount of DSF were used as controls. Samples of different times were treated at regular intervals as described above. Metabolites were detected by gas chromatography–mass spectrometry (GC–MS) (Model 7890B/5977B, Agilent Technologies Inc., Santa Clara, CA, USA). To identify the degradation products, mass spectrometry analyses were compared with the authentic standard compounds of the National Institute of Standards and Technology (NIST, Gaithersburg, MD, USA) library database.

The analytical conditions for GC–MS were as follows: injection amount, 1.0 μL; injection mode into the gas chromatograph, split with a 10:1 ratio; capillary column, HP-5 MS Ultra Inert column (30 m\0.25 μm\ 0.25 μm, Agilent Technologies Inc., Santa Clara, CA, USA); column oven temperature, 60 °C for 5 min, then an increase of 10 °C/min, followed by 300 °C for 29 min; injection port temperature, 280 °C; carrier gas, helium; analytical mode, scan mode. Mass spectra were recorded in the *m*/*z* range of 40–430.

### 2.7. Biocontrol Assay of Strain HN-2 Against Xcc

A biocontrol assay was carried out on slices of radish root (*Raphanus sativus*) [23]. Plants purchased from a local market were thoroughly washed under running tap water. Surface sterilization of plants was carried out by immersing plants sequentially in 44% sodium hypochlorite and 70% ethanol for 1 min followed by rinsing in sterile distilled water (D/W). The plant sterilization procedure was repeated three times, and slices of radish root 4–5 mm in thickness were prepared. *Cupriavidus* sp. HN-2 (9.6 × 10^7^ CFU·mL^–1^) isolate was mixed with XC1 (4 × 10^8^ CFU·mL^–1^), and 100 μL of the mixture was inoculated on each slice. Slices were placed in sterile plates and incubated at 28 °C for 2 d; the diameter of the macerated region was considered as the lesion diameter (mm). Macerated tissue was scooped out and its weight was noted to compare with the initial weight of the tissue before inoculation, and maceration percentage was calculated. Five control groups included pure HN-2, pure XC1, XC1 mixed with *E. coli* DH5α (8 × 10^8^ CFU/mL), XC1 mixed with agricultural streptomycin, and D/W, respectively. Three independent trials were carried out in triplicate.

### 2.8. Data Analysis

Experiments were arranged as completely randomized designs with three replicates. Data were analyzed by one-way analysis of variance (ANOVA), and means were compared according to Bonferroni’s multiple comparisons test using Graphpad Prism software (version 6). Statistical significance was determined by Tukey’s honest significance difference (HSD) test at *p* < 0.05 to examine specific differences between groups.

### 2.9. GenBank Accession Numbers

The 16S rDNA gene sequence of isolate *Cupriavidus* sp. HN-2 was submitted to GenBank (GenBank accession No. MG561941.1).

## 3. Results

### 3.1. Isolation, Screening, and Identification of DSF-Degrading Isolates

In this study, a simple and efficient method was developed to effectively screen DSF-degrading bacteria. According to the results of the soil enrichment culture, four morphologically different bacterial strains were attained by the streaking plate method and named as HN-1, HN-2, HN-3, and HN-4, respectively. The bacterial strains HN-1, HN-2, HN-3, and HN-4 degraded DSF 81.0%, 100%, 79.9%, and 69.0% within 24 h of incubation, respectively (Table S1). The results showed that HN-2 possessed the highest degradation capacity among the four strains. Strain HN-2 completely degraded 2 mM DSF within 24 h of incubation. In addition, the inhibition test indicated that strain HN-2 showed no inhibitory activity against *Xcc* (Figure S1). Thus, strain HN-2 was selected for further studies.

**Figure 3:**
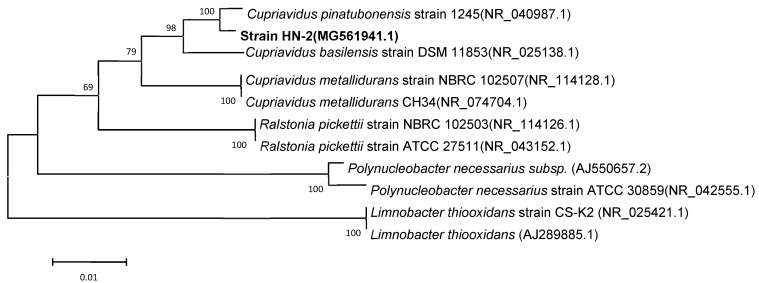
Phylogenetic tree based on the 16S rDNA sequence of *Cupriavidus* sp. strain HN-2 (represented in bold) and related strains. Numbers in parentheses represent the sequence accession numbers in GenBank. Numbers at the nodes indicate the bootstrap values from neighborhood-joining analysis of 1000 re-sampled data sets. Bars represent sequence divergence.

Light and scanning electron microscopy revealed that strain HN-2 is an aerobic, asporogenic, Gram-negative bacterium; it is ellipse to rod-shaped (0.4–0.8 × 0.6–1.2 μm), straight, and is without flagella (Figure S2). Strain colonies appear as circular, opaque, convex, and white on an LB plate. The cellular properties and colony morphology of strain HN-2 were similar to *Cupriavidus pinatubonensis*. According to BLAST results of the 16S rDNA sequence on the NCBI, strain HN-2 showed high similarity (≥99%) with different *Cupriavidus* strains (Table S2). Strain HN-2 was closely clustered with the *Cupriavidus* group in a phylogenetic tree and therefore was classified as a *Cupriavidus* sp. (Figure 3). *Cupriavidus* sp. strain HN-2 isolated in this study was deposited in the Guangdong Microbial Culture Center (CDMCC) under CDMCC 60432.

### 3.2. DSF Degradation Kinetics

**Figure 4:**
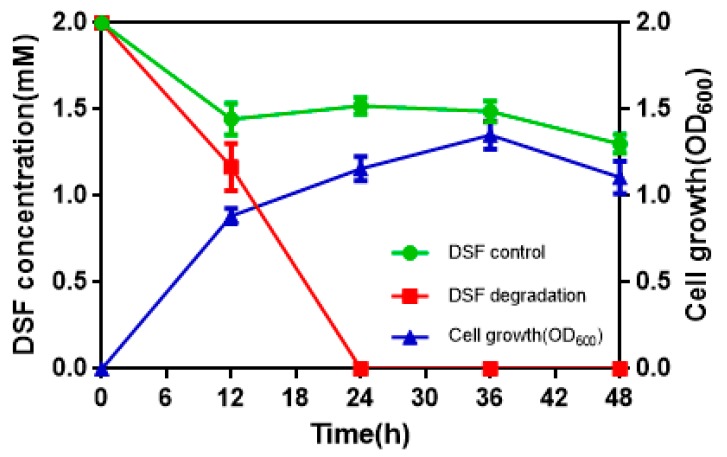
The relationship between diffusible signal factor (DSF) degradation and growth of strain HN-2. Values represent the mean of three trials. Each trial has three replicates. Bars indicate standard deviation of the mean.

To determine the quantitative differences between DSF degradation and HN-2 growth, DSF degradation was measured in vitro. Figure 4 presents the relationship between DSF degradation and growth of HN-2. DSF degradation was 42.1%, 100%, 100%, and 100% after 12 h, 24 h, 36 h, and 48 h, respectively. During the fastest growing period (0–12 h), the OD_600_ reached 0.840, and the DSF concentration decreased from 2.0 to 1.158 mmol·L^–1^. DSF was completely degraded within 24 h (Figure 4, Figure S3). In contrast, degradation of the control group (without strain HN-2) was significantly lower and reached only 10% after 24 h.

### 3.3. Degrading Products and Degradation Pathway of DSF

Samples obtained at different times were analyzed through GC–MS to identify the degradation products during the bacterial degradation process. In the initial sample of the 0 h and 2.0 mM DSF standard, a significant peak was detected at retention time (RT) of 16.972 min, displaying a characteristic mass fragment [M+] at an m/z value of 99.04, with major fragment ions at m/z values of 43.06 and 152.15 (Figure S4a), which were identified as DSF. Subsequently, the DSF disappeared concomitantly with the formation of a new compound with an m/z value of 112.99 eluted at 16.977 min. According to the similarity of the fragment retention times and molecular ions to those of the corresponding authentic compounds in the NIST library database, the compound was characterized as trans-2-decenoic acid methyl ester (Figure S4b). Finally, DSF was completely degraded by strain HN-2 without any persistent accumulative product.

**Figure 5:**
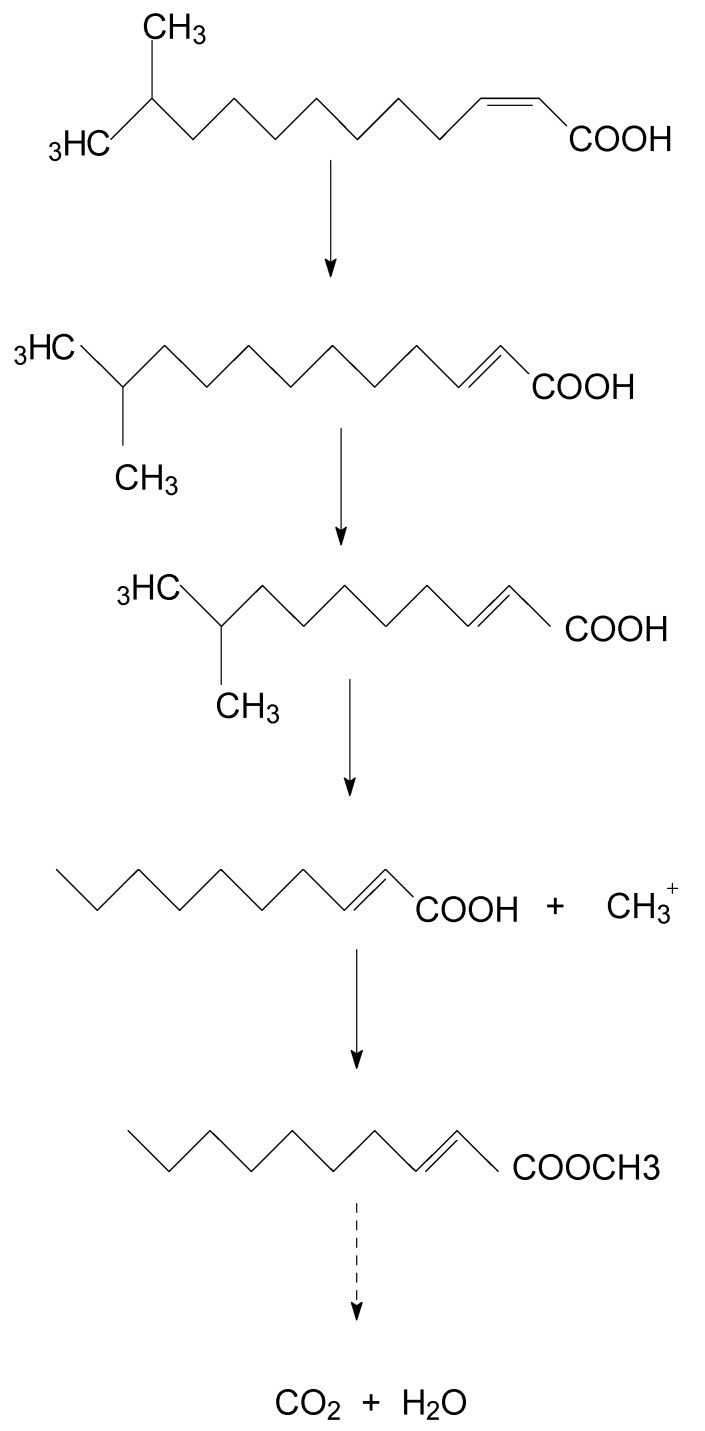
Proposed degradation pathway of DSF in strain HN-2.

A new degradation pathway of DSF in strain HN-2 was proposed based on the chemical structures of DSF and the most probable degradation product formed during the metabolic process (Figure 5). First, the cis double bond of DSF was converted to a trans double bond by isomerase. Then, through β-oxidation of the fatty acid, trans-11-methyl-2-dodecenoic lost two carbons and β-oxidation stopped. The intermediate product lost a methyl group to produce a free methyl group and trans-2-decenoic acid. Then, the free methyl group replaced the hydrogen on the carboxyl group of trans-2-decenoic acid to form trans-2-decenoic acid methyl ester.

### 3.4. Biocontrol Potential of Isolate HN-2

**Figure 6:**
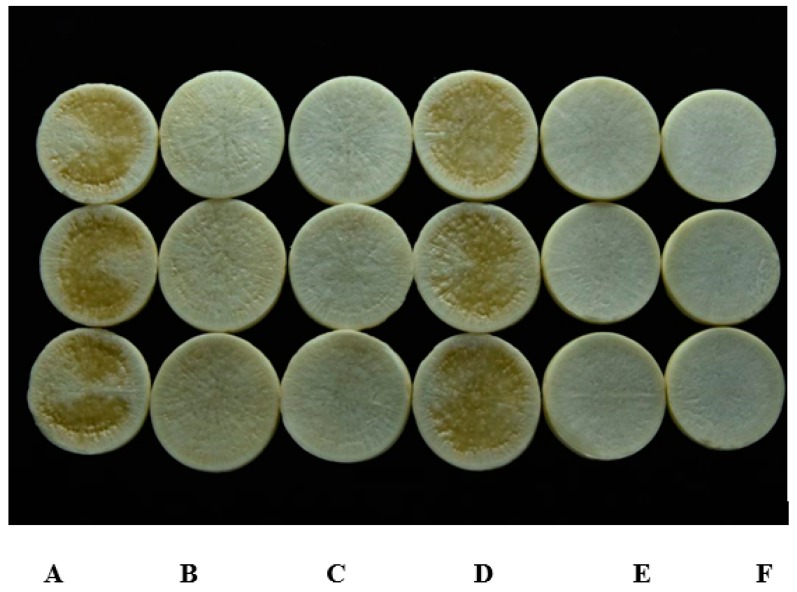
Biocontrol test of strain HN-2 against black rot disease on radish slices under laboratory conditions. (**A**) *Xcc* XC1 inoculated alone on radish. (**B**) *Xcc*XC1 + HN-2. (**C**) *Xcc*XC1 + agricultural streptomycin. (**D**) *Xcc*XC1 + *E. coli* DH5α. (**E**) HN-2 inoculated alone on radish. (**F**) Distilled water (D/W) inoculated on radish as control.

**Figure 7:**
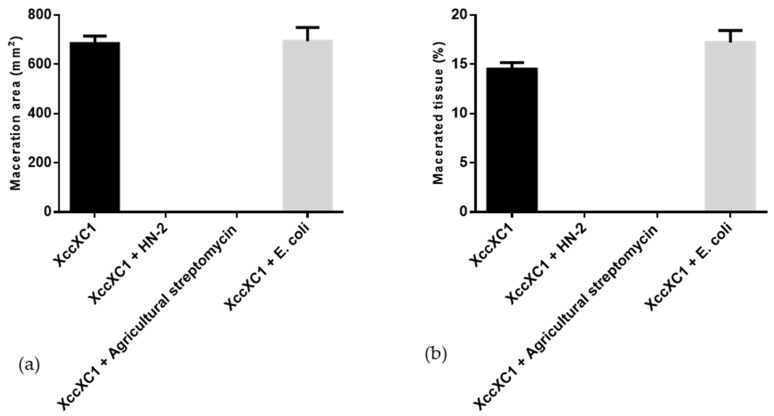
Quantification of radish tissue maceration. (**a**) Maceration area. (**b**) Macerated tissue. Values represent the mean of three trials. Each trial has three replicates. Bars indicate standard deviation of the mean. Statistical significance determined at *p* < 0.05 according to the Tukey’s honest significance difference (HSD) test.

Strain HN-2 showed 100% biocontrol efficiency against *Xcc* on radish slices at a concentration of 9.6 × 10^7^ CFU·mL^–1^ (Figure 6). Plant slices treated solely with XC1 or an *E. coli* DH5α and XC1 mixture resulted in severe incidence of disease (Figure 6A,D). Individual treatment of strain HN-2 significantly reduced disease severity caused by *Xcc*. Maceration was not observed in plant slices treated with a mixture of HN-2 and XC1 (Figure 6B and Figure 7), similar to the treatment of agricultural streptomycin and the XC1 mixture (Figure 6C and Figure 7). A biocontrol agent should not harm the host plant, and results of isolate HN-2 inoculation on surface-sterilized slices proved it safe for further use (Figure 6E). This study confirms the efficient DSF degrading bioactivity of isolate HN-2 against the pathogen XC1 and presents it as a potential biological tool to control DSF-dependent bacterial pathogens.

## 4. Discussion

Bacteria employ different and efficient biological tools to manage bacterial and fungal plant diseases. However, only a few of them can rapidly degrade and inactivate the quorum sensing signal molecule DSF. In a search for naturally occurring biological control agents, a bacterial strain *Cupriavidus* sp. HN-2 from agricultural contaminated fields was screened for its biocontrol potential against *Xcc*. Moreover, few studies are available about the potential biocontrol activity of *Cupriavidus* sp. against *Xcc*. This study presents the first evidence that *Cupriavidus* sp. can substantially reduce the severity of black rot disease caused by *Xcc*.

QQ has recently been recognized as a potential new strategy for disease control [24,25,26]. Several Gram-negative pathogenic bacteria, including *Agrobacterium*, *Brucella*, *Burkholderia*, *Enterobacter*, *Pectobacterium*, *Pseudomonas*, *Ralstonia*, *Serratia*, *Vibrio*, and *Yersinia*, utilize QS to regulate their virulence, makes QS an emerging target for QQ biocontrol agents to control phyto-pathogens [23,27,28]. QQ strategies do not aim to kill pathogens but rather affect the expression of a specific function regulated by QS; therefore, they theoretically exert less selective pressure than biocide treatments. This makes QQ a valuable strategy to develop sustainable biocontrol agents and avoid antibiotic resistance [15,20,21,29,30,31,32]. In this regard, potential DSF degraders belonging to the genera *Bacillus*, *Acinetobacter*, *Paenibacillus*, *Microbacterium*, and *Staphylococcus* were isolated [27,28,33]. In the present study, a novel DSF-degrading isolate, HN-2, efficiently degraded the DSF signaling molecule of XC1 without affecting its growth. In addition, with the sequencing of the genome of the HN-2 strain, finding the gene clusters and enzymes responsible for DSF degradation may reveal the metabolic pathway for complete degradation and metabolism of DSF. We hypothesized that the cis double bond of DSF would be converted to a trans double bond by an isomerase. Then, through β-oxidation of the fatty acid, trans-11-methyl-2-dodecenoic lost two carbons, and β-oxidation did not continue. Catalyzed by an enzyme, the intermediate product lost a methyl group to produce a free methyl group and trans-2-decenoic acid. Then, the free methyl group replaced the hydrogen on the carboxyl group of trans-2-decenoic acid to form trans-2-decenoic acid methyl ester. Oxidation and hydroxylation play significant roles in the biodegradation of DSF.

In addition to its DSF-degrading capability, the biocontrol effects of the HN-2 isolate were also evaluated. Application of strain HN-2 as a biocontrol agent could substantially reduce the disease severity of *Xcc*. Antibiotic-producing *Pseudomonas fluorescens* could protect crops from root pathogens but its negative impact on the development of tomato roots limits its utility in the biological control of pathogens [34]. Interestingly, isolate HN-2 tested during the current study did not adversely affect the host plants. Potential biocontrol species must not be harmful to the host plants; therefore, any signal-molecule-degrading bacterial species cannot be used as a biological control agent. Similar to other pathogens, XC1 also has a broad host range and can infect plants of different families, including *Brassicaceae* (radish). During the bioassays, isolate HN-2 not only survived on radish but also attenuated black rot symptoms. These characteristics reveal the potential of isolate HN-2 as a broad-range biocontrol agent and especially prove its utility in controlling the infectious diseases caused by DSF-dependent bacterial pathogens.

## 5. Conclusions

In this study, a novel quorum quenching bacterial isolate, *Cupriavidus* sp. HN-2, exhibiting potent biocontrol potential against *Xcc*, was identified. Strain HN-2 has superb DSF degradation activity and harbors the metabolic pathway for complete degradation and metabolism of DSF. Moreover, the application of strain HN-2 as a biocontrol agent could substantially reduce the disease severity of *Xcc*. These findings reveal the biochemical basis of a highly efficient DSF-degrading bacterial isolate and identify useful agents that exhibit the potential for biocontrol of infectious diseases caused by DSF-dependent bacterial pathogens. However, to develop an effective biological control agent, the bacterial strain should be comprehensively studied and optimized before field applications. An in-depth study of the gene clusters and enzymes responsible for DSF degradation by the sequencing of the genome of HN-2 strain will provide insights into the degradation mechanisms.

## Supplementary Materials

The following are available online at https://www.mdpi.com/2076-2607/8/1/45/s1.
