# Supplementary material for: Cupriavidus sp. HN-2, a Novel Quorum Quenching Bacterial Isolate, is a Potent Biocontrol Agent Against Xanthomonas campestris pv. campestris
Source: Microorganisms. 2019 Dec 25;8(1):45. doi: 10.3390/microorganisms8010045 (PMC7022395; doi:10.3390/microorganisms8010045)
Supplement: Supplementary file 1 [file microorganisms-08-00045-s001.pdf]

Supplementary Materials

Cupriavidus sp. HN-2, a Novel Quorum Quenching Bacterial Isolate, is a Potent Biocontrol Agent Against *Xanthomonas campestris* pv. *campestris*

Tian Ye [1,2,†], Tian Zhou [1,2,†], Qiting Li [1,2,†], Xudan Xu [1,2], Xinghui Fan [1,2], Lianhui Zhang [1,2] and Shaohua Chen [1,2,*]

[1] State Key Laboratory for Conservation and Utilization of Subtropical Agro-bioresources, Guangdong Laboratory of Lingnan Modern Agriculture, Integrative Microbiology Research Centre, South China Agricultural University, Guangzhou 510642, China; 20182047012@stu.scau.edu.cn (T.Y.); 20171021009@stu.scau.edu.cn (T.Z.); 201614090409@stu.scau.edu.cn (Q.L.); xuxudan@stu.scau.edu.cn (X.X.); fxhscau@163.com (X.F.); lhzhang01@scau.edu.cn (L.Z.)

[2] Guangdong Province Key Laboratory of Microbial Signals and Disease Control, South China Agricultural University, Guangzhou 510642, China

* Correspondence: shchen@scau.edu.cn; Tel+86-20-85288229

† These authors contributed equally to this work.

Table S1. Isolation and screening of DSF-degrading strains.

Strains	DSF degradation (%)
HN-1	81.0 ± 0.9
HN-2	100 ± 0.2
HN-3	79.9 ± 0.6
HN-4	69.0 ± 1.0

Note: Means are averaged values of three replicates.

Table S2. The BLAST results of different *Cupriavidus* strains were detected at >99% similarity.

Cupriavidus sp.	Query Cover	E value	Similarit y	Accession
Cupriavidus sp. strain HN-2	100%	0	100.00%	MG561941.1
Cupriavidus sp. PND-6	99%	0	99.86%	EF687003.1
Cupriavidus sp. TFD45	99%	0	100.00%	EU827492.1
Cupriavidus sp. SM-2017	99%	0	99.71%	LC218379.1
Cupriavidus pinatubonensis strain: 1266	99%	0	99.50%	AB121227.1
Cupriavidus pinatubonensis strain: 1243	99%	0	99.50%	AB121220.1
Cupriavidus pinatubonensis strain: 1247	99%	0	99.43%	AB121223.1
Cupriavidus pinatubonensis strain 1245	99%	0	99.43%	NR_040987.1
Cupriavidus pinatubonensis strain: 1249	99%	0	99.36%	AB055427.2
Cupriavidus sp. PIC4	99%	0	99.50%	KF954774.1
Cupriavidus pinatubonensis type strain CCUG53907T	98%	0	99.64%	FN597611.1

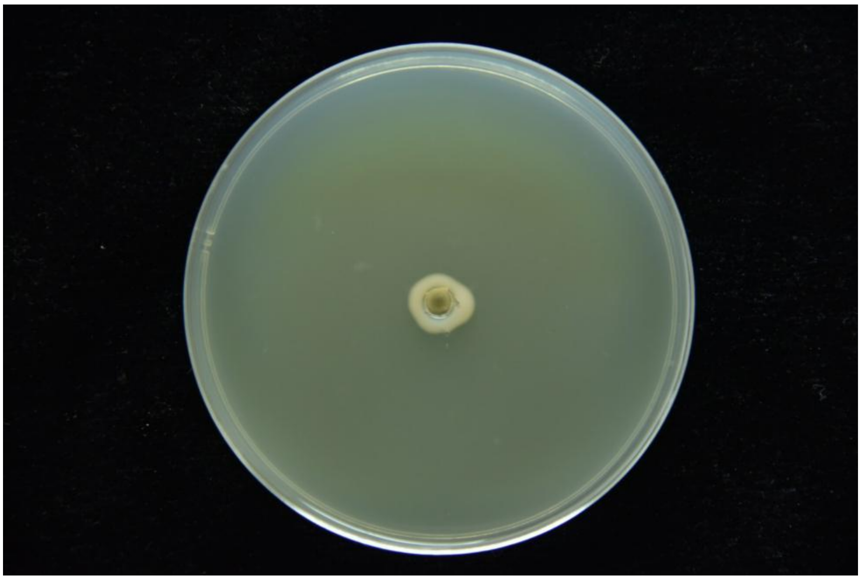

Figure S1. The isolate HN-2 did not show antagonism against *Xcc*. Holes punched in the LB plate contained culture solution of *Xcc* and isolate HN-2.

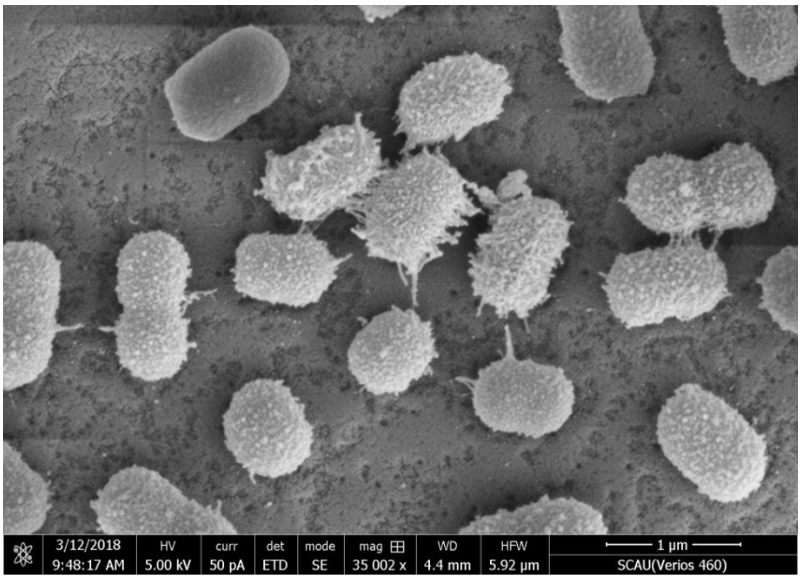

Figure S2. The scanning electron microscope image of strain HN-2. The strain HN-2 cell was ellipse to rod-shaped (0.4–0.8 × 0.6–1.2 μm), straight, and without flagella.

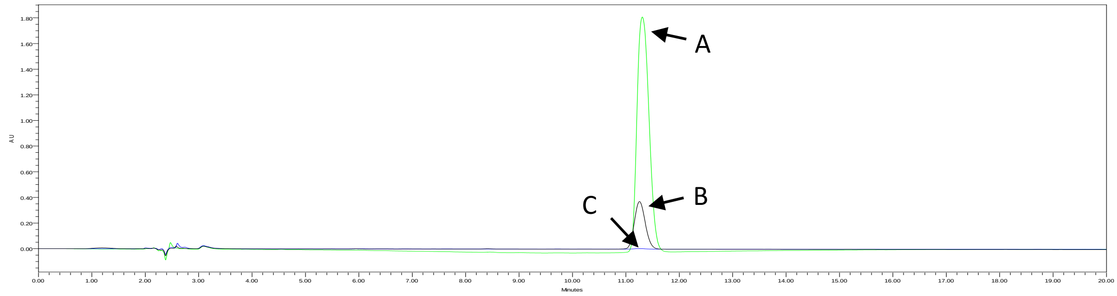

Figure S3. The remaining amount of DSF at different time points determined by HPLC. A, B, C represent the amount of remaining DSF with the strain HN-2 at 0 h, 12 h, and 24 h.

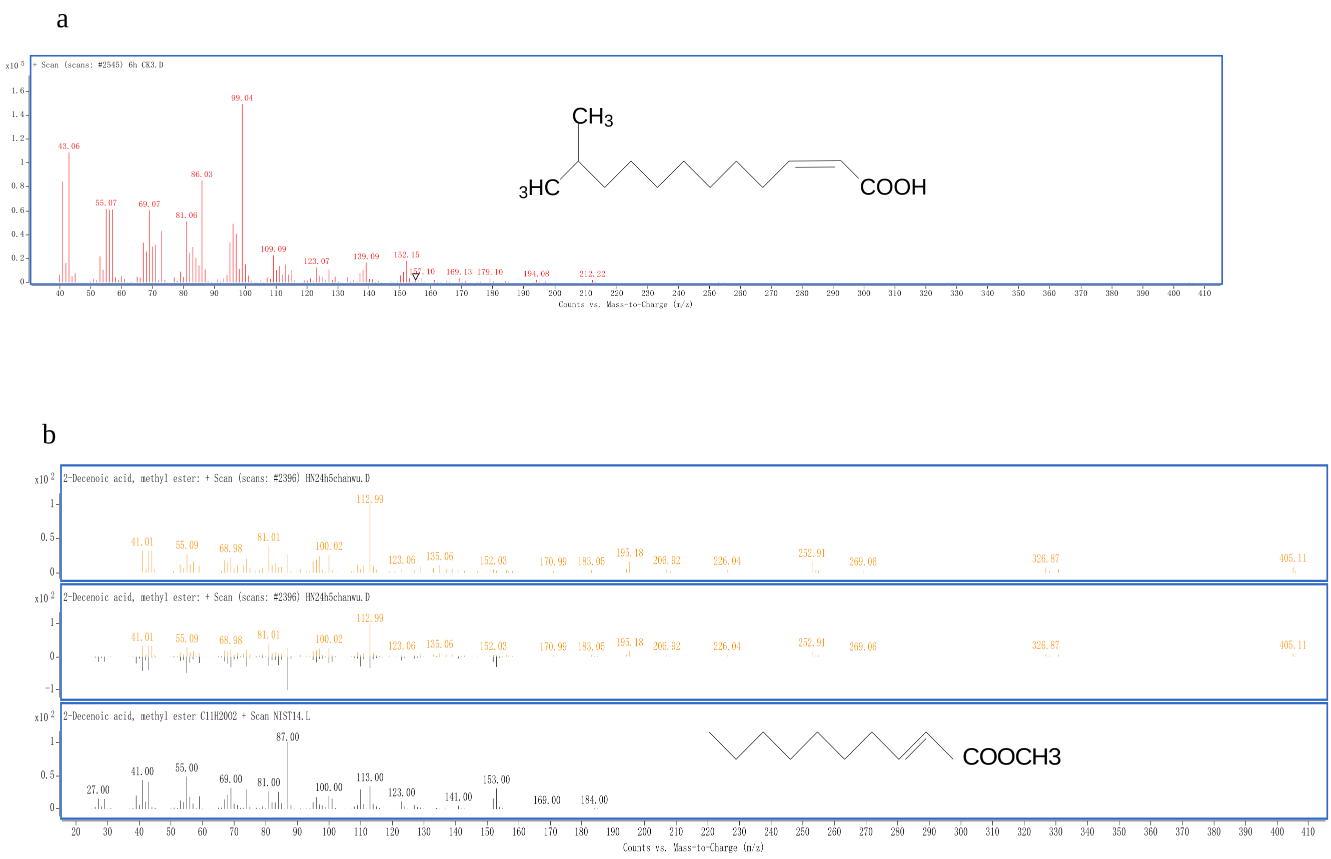

Figure S4. Mass spectra and proposed structures of detected degradation products identified with GC–MS. (a) DSF; (b) *trans*-2-decenoic acid methyl ester.
